# Buffering the effects of bad news: Exposure to others’ kindness alleviates the aversive effects of viewing others’ acts of immorality

**DOI:** 10.1371/journal.pone.0284438

**Published:** 2023-05-17

**Authors:** Kathryn Buchanan, Gillian M. Sandstrom

**Affiliations:** 1 Department of Psychology, University of Essex, Essex, United Kingdom; 2 Department of Psychology, University of Sussex, Brighton, United Kingdom; Birla Institute of Technology and Science, Pilani, INDIA

## Abstract

Negatively valenced news dominates the press, with stories of death and destruction gaining significant traction while also negatively impacting people’s mental health and perceptions of humanity. Given that horrific acts happen and need to be reported, we examined if news stories featuring others’ kindness could undo the aversive effects of news stories featuring others’ immorality. In Studies 1a-d we tested whether media exposure to the acts of kindness that occurred in response to a terrorist attack could alleviate the aversive effects of media exposure to the terrorist attack. In Study 2, we examined whether, more generally, the aversive effects of news stories featuring immorality (e.g., homicide, paedophilia, bullying) could be alleviated through news stories featuring acts of kindness (e.g., volunteering, philanthropy, caring for the homeless). In Studies 1 and 2, we found that participants exposed to others’ immorality and then others’ kindness suffered from less aversive changes to their mood, experienced greater levels of elevation and were more inclined to believe in the goodness of others, than participants exposed only to others’ immorality. Given this, we suggest there is merit in journalists shining a light on others’ kindness if people’s affective well-being and belief in the goodness of humanity is to remain intact.

## Introduction

Journalists employing the maxim “if it bleeds, it leads” seem intuitively aware of the negativity bias people have in attending to and remembering bad events over good ones [1]. Indeed, negatively valenced news dominates the press [2] and is shared on Twitter more frequently than positively valenced news [3]. Similarly, tragedies such as mass shootings and terrorist atrocities are the most closely followed news stories [4]. Yet, while news featuring others’ immorality captivates people, it can have aversive affective and cognitive impacts, increasing emotional disturbances and negatively skewing people’s belief in the goodness of others [5–7]. Given that bad things happen and need to be reported, we consider whether anything can be done to alleviate the aversive effects of negatively valenced news on news recipients. Accordingly, for the first time, we examine if the aversive effects of media exposure to immorality (e.g., terrorism, paedophilia, homicide) can be alleviated by subsequent exposure to news stories featuring kindness.

### Negative effects of negatively valenced news

The omnipresence of negatively valenced news afforded by a global pandemic has led several researchers to convincingly document its adverse effects on mood and mental health. Specifically, cross-sectional studies using robust sample sizes have repeatedly linked exposure to COVID-related news to poorer mental health, recording higher levels of worry, hopelessness, distress, anxiety and depression [8, 9]. Similarly, we found in our previous work that just 2–4 minutes of exposure to COVID-related news detrimentally impacted people’s emotional well-being compared to a no-treatment control condition [10]. The deleterious effects of negatively valenced news are not unique to the pandemic but were also documented in response to the 9/11 terror attacks. For instance, short term exposure to media coverage of the event triggered the prevailing responses of shock, fear, and anger [6, 11, 12], while longer term exposure predicted increased incidences of PTSD, even in those not directly affected by the attacks [7, 13–15]. Pandemic and terrorist incidences aside, researchers have also found that the general consumption of negatively valenced news can lead to emotional disturbances such as sadness and anxiety, and maladaptive thought patterns including increased pessimism and catastrophizing [5, 16, 17]. As such, there is a clear indication that negatively valenced news has negative effects on mood and mental health [see also, 18].

The aversive effects of negative news stories are not just limited to mood disturbances. Researchers have found that exposure to negatively valenced media can trigger compassion fatigue, the reinforcement of racist stereotypes, and a belief that the world is a dangerous place where people cannot be trusted [19–22]. Furthermore, when people are exposed to negative information about others it can promote political apathy [23] and demotivate people from undertaking positive social actions such as philanthropy and eco-orientated actions [5]. Indeed, such findings have led to calls for a move towards constructive journalism, which aims to spurn negativity bias in favour of a more accurate portrayal of real-world issues that includes context, progress and solutions [24].

### Witnessing others’ kindness

Ample research has consistently established that media exposure to others’ immorality impacts people negatively, causing aversive affective and cognitive responses. Far less is known about whether media exposure to others’ kindness can impact mood and perceptions of society, or whether it can subsequently alleviate the aversive affective and cognitive responses caused by media exposure to others’ immorality. On the one hand, there is cause for pessimism given that the effects of bad are theorized to be stronger than that of good [1]. This so called negativity bias [25], is evident in research findings that negative information is attended to and processed more deeply than positive information [26]. Importantly, given the focus of our study, researchers have convincingly demonstrated the negativity bias in over 6 continents, by showing that on average people experience stronger physiological reactions to real-world negative news stories, than positive ones [27].

On the other hand seeing others’ kindness may prove powerful in a) enabling people to maintain core beliefs that others are good, b) providing a resolution to seeing others’ suffering, and c) eliciting elevation: a positive and uplifting feeling that occurs in response to seeing acts of moral beauty and is theorized to have restorative properties for affective and cognitive well-being [28–30].

### We value kindness and want to believe the world is good

Benevolence is consistently rated as an important value across numerous countries [31, 32], and generally people appear motivated to believe in the goodness of others [33] and to evaluate humans and their nature positively [34]. For instance, in two nationwide convenience samples, scores for belief in pure good were substantially larger than scores for belief in pure evil [35]. Similarly, children and adults alike view good in more essentialist terms than bad [36].

Believing in others’ goodness appears to be beneficial for people, as positive perceptions of humanity have been significantly correlated with various indicators of well-being [34, 35, 37–40], while experimental research has found that simply noticing others’ acts of kindness can increase happiness [41]. Conversely, shattered assumption theory suggests when a trauma alters our beliefs about the goodness of others we experience post-traumatic distress [42].

Clearly, our belief in the goodness of others can be threatened by media exposure to others’ immorality [6, 21]. For instance, mean world syndrome describes the phenomenon whereby people perceive the world to be more dangerous than it really is, due to repeated and consistent media exposure to violence-related content [43]. Yet seeing others’ kindness may allow people to maintain a core belief that is important for well-being—that overall the world, and the people in it are good [33].

### Seeing others helped is the resolution to seeing others hurting

One striking aspect of disasters is that they invoke prolific responses of altruism and generosity [44]. Indeed, so called “catastrophe compassion”–positive behaviour under negative circumstances [45]–has been well documented. For example, in response to the COVID-19 pandemic, data from the World Happiness Report evidenced substantial increases in pro-sociality in 2020–2021, with willingness to help strangers doubling since 2018–2019, alongside significant increases in donations and volunteering [46]. Similarly, in the aftermath of 9/11 the public responded with pro-social behaviour in various forms including general empathic responses, blood donations and charitable gifts to victims affected by the attack [12].

Catastrophe compassion is caused by empathy [45], a known motivator of pro-sociality [47, 48]. Indeed, initial research suggests that from an early age, humans have a genuine concern for the welfare of others [49]. It seems, when we see others hurt it hurts us too [50], and we want to help. Helping can prove a fruitful coping mechanism in stressful situations [51, 52]. For instance, during the first COVID-19 pandemic lockdown, pro-social behaviour was consistently associated with better well-being across eight samples from various countries [53]. In general, a considerable body of literature has documented that we experience greater subjective well-being when we help others [54, 55]. For instance, one study found that trying to make others feel good was a more effective happiness enhancing strategy than trying to make oneself feel good [56]. However, it is not yet known whether observing others being helped, is as beneficial for well-being as being the one to help others out.

We anticipate that in situations where people experience empathy and compassion in response to others’ adversity but are unable to personally help, they will still experience relief or satisfaction that others are being helped; seeing others being helped should provide resolution to seeing others hurt, whether or not they are the ones directly helping them. In line with this, researchers have observed reductions in participants’ physiological arousal when witnessing a harmed victim being helped [57]. Similarly, even those who have not directly helped out in the wake of a disaster can benefit from witnessing catastrophe compassion as it benefits communities through bolstering social connection, solidarity, and shared resilience [45].

### Seeing others’ kindness elicits elevation, which has restorative capabilities

Beyond being a common response to a crisis, kindness is also a powerful elicitor of elevation [58]–a multifaceted construct comprising emotional, cognitive and physiological responses that involves feeling uplifted, experiencing warmth in one’s chest, wanting to be a better person and feeling optimistic about humanity [59, 60]. Conceptually and theoretically distinct from more generalised positive affect (e.g., joy, happiness) [28], elevation has been referred to as an “emotional reset” button that has the potential to reduce “feelings of cynicism…and replace them with feelings of hope, love and optimism” [29, p.286]. Given that kindness elicits elevation, which is theorised to have a restorative functionality, we anticipated that exposure to others’ kindness may alleviate the emotionally aversive effects of exposure to others’ immorality.

## Current research

The primary aim of the present research was to test the proposition that witnessing others’ acts of kindness would alleviate some of the unpleasant feelings and cognitions experienced after media exposure to others’ acts of immorality. Specifically in Studies 1a-d we tested whether media exposure to acts of kindness that occurred in response to a terrorist attack could alleviate the aversive effects of media exposure to a terrorist attack. In Study 2, we aimed to increase the generalisability of our findings, and focused more generally on whether news stories featuring kindness (e.g., volunteering, philanthropy, caring for the homeless) could alleviate the effects of news stories featuring immorality (e.g., homicide, paedophilia, bullying).

## Studies 1a-d

In Studies 1a-d we measured participants’ positive and negative affect before and after they were randomly assigned to view media footage of either a terrorist incident on its own, or a terrorist incident plus the kindness enacted by the general public in response to a terrorist incident. In line with past research, we expected that media exposure to the terrorism would result in decreases in positive affect and increases in negative affect, relatively lower levels of elevation, and more negative perceptions of humanity (vs. before the media exposure). However, we expected that if this was followed by viewing others’ acts of kindness, it would help to attenuate the negative impacts on a) affect, b) elevation and c) perceptions of humanity.

## Method

### Ethics approval

Prior to commencement of this research, Studies 1a-d were reviewed by the Faculty of Science and Health Ethics Committee at the University of Essex and granted approval with the following code: KB1702. We did not request, and were not granted approval to conduct research with participants under the age of 18. However, in Study 1a, 7 respondents reported they were aged < 18, consequently we did not include their data in our analysis. In Studies 1a-d, survey respondents provided explicit “written” consent to participate in the online study by selecting the response button “I agree to give my consent to participate in this research”.

### Study design

Studies 1a-d varied slightly in their design (see Table 1) but, in general, participants were randomly allocated to one of four possible conditions: Immorality, Kindness, Immorality+Kindness, and Control. While the content shown varied depending on the condition they were assigned to, across all studies and all conditions, participants were shown video clips of between 1–3 minutes. The Immorality condition involved participants watching media footage of a UK-based terrorist attack that had occurred between 2–7 months prior to these studies. The Kindness condition, which was not included in Study 1a, involved participants watching media footage of 5 kind acts performed by the British public in response to the same terrorist attack. The Immorality+Kindness condition involved participants watching both the terrorist attack clip and the kindness clip. The Control condition was designed to produce a neutrally affective state, however this proved challenging to achieve, hence the Control condition varied between studies.

**Table 1:** Studies 1a-d: Summary of study designs.

Study	Condition	Clip Viewed
Study 1a	Control	Baking tutorial. https://tinyurl.com/yxqydcyr
	Immorality	Ariana Grande concert bombing (Manchester, UK, May 2017) https://tinyurl.com/y37yjcsa
	Kindness	[N/A Not included].
	Immorality+Kindness	Ariana Grande concert bombing & footage of 5 kind acts that occurred in the wake of the concert bombing https://tinyurl.com/y2fkujtt
Study 1b	Control	Outdated weather forecast featuring sun and showers (UK, October, 2016) https://tinyurl.com/y3g3gecg
	Immorality	As per Study 1a.
	Kindness	As per the kindness part of the Immorality+Kindness condition in Study 1a.
	Immorality+Kindness	As per Study 1a.
Study 1c	Control	As per Study 1b.
	Immorality	London Bridge terrorist attack (London, UK, July 2017) https://tinyurl.com/mryd7x75
	Kindness I	As per Study 1b.
	Kindness II	5 kind acts that occurred in response to the London Bridge terrorist attack. https://tinyurl.com/y9hxpr7j
	Immorality+Kindness II (matched)	London Bridge terrorist attack & 5 kind acts that occurred in response to it
	Immorality+Kindness I (unmatched)	London Bridge terrorist attack & 5 kind acts that occurred in response to the Ariana Grande concert bombing.
Study 1d	Control	PowerPoint presentation featuring changing shapes. Participants asked to count number of circles. https://tinyurl.com/yxbqlmsc
	Immorality	As per Study 1a.
	Kindness	As per Studies 1b, and 1c (Kindness I).
	Immorality+Kindness	As per Studies 1a, 1b, and 1c (Matched).
	Amusement	A pilot drawing a Christmas tree. https://tinyurl.com/cb62h3pd
	Immorality+Amusement	Ariana Grande concert bombing & pilot drawing a Christmas tree.

Notably, Study 1c differed in its design as it sought to examine whether kindness needs to be related to the immorality in order to alleviate the emotional impact of it–hence the inclusion of matched and unmatched Immorality+Kindness conditions, and the addition of a second kindness clip. However, subsequent analyses found the matched and unmatched conditions had a similar impact on changes to affect (for positive affect: matched *t*(40) = -1.55, *SE* = .11, *p* = .129, CI95 = [-.56, .07]; unmatched *t*(34) = -0.11, *p* = .911, CI95 = [-.27, .24]; for negative affect: matched *t*(39) = 5.29, *SE* = .09, CI95 = [.31, .69] and unmatched *t*(df) = 4.52, *SE* = .11, CI95 = [.27, .72], both *p*’s < .001). The two Kindness clips also had similar impacts of changes to affect, in that both significantly decreased negative affect (Kindness I *t*(47) = 3.68, *SE* = .11, CI95 = [.18, .62], Kindness II *t*(41) = 3.65, *SE* = .08, CI95 = [.12, .43], both *p*’s < .001) and increased positive affect (Kindness I *t*(47) = 1.45, *SE* = .09, *p* = .154, CI95 = [-.05, .32]; Kindness II *t*(42) = 2.29, *SE* = .11, *p =* .027, CI95 = [.03, .49]). Consequently, we combined a) the matched and unmatched immorality kindness conditions (Immorality+Kindness) and b) the two kindness conditions (Kindness) to streamline analyses.

Finally, Study 1d’s design differed from Studies 1a-c as it included the additional conditions of Amusement, and Immorality+Amusement. These were included to test whether the emotional impacts of seeing others’ immorality could be alleviated by any clip that induced positive affect, or whether the effect was specific to kindness.

### Stimuli

Stimuli can be accessed via the links included in Table 1. To increase ecological validity, we used genuine clips of news reports that were accessed via popular UK media channels (i.e., the BBC, ITV, The Guardian, Huffington Post).

### Participants

Between August 2017 and January 2018 data were collected for four studies from four different samples (Study 1a: *N* = 211, Study 1b: *N* = 180, Study 1c: *N* = 240, Study 1d: *N* = 509). A post-hoc sensitivity analysis indicates that all four studies provided adequate power (>.80) to detect a small effect size (partial η^2^ = 0.02).

In Studies 1a and 1c, respondents were recruited using a convenience sampling methodology and received no payment for their participation. In Studies 1b and 1d, all respondents received a nominal fee in return for their participation and were recruited via Prolific Academic (Study 1b), and Amazon’s Mechanical Turk (Study 1d). Respondents’ data were not included in the final samples if they were aged 18 or under (as per the conditions of our ethics), failed one or more attention checks, reported not watching the video clips in full or whose hidden page timer indicated they had progressed with the survey without watching the video clips in full. In addition, to achieve homogenous samples in Studies 1a, b and c which utilized a British sample, participants were excluded if they were not UK residents, and in Study 1d which utilized an American sample, participants were excluded if they were not US residents. The sample characteristics and the exclusion criteria are detailed in Table 2.

**Table 2:** Studies 1a-d: Sample characteristics.

	Study 1a	Study 1b	Study 1c	Study 1d
***Sample size***				
Initial sample size	289	210	388	644
Final sample size	211	180	240	509
***Sample size per condition***				
Control	70	44	38	90
Immorality	76	47	37	90
Kindness	-	44	91	86
Immorality+Kindness	65	45	74	72
Amusement	-	-	-	89
Immorality+Amusement	-	-	-	82
***Exclusion reason***				
Under 18	7	-	-	-
Not UK Resident	18	3	70	-
Not US Resident	-	-	-	5
Failed one or more attention checks^1^	-	3	14	4
Reported not watching clips in full^2^	21	4	28	21
Page timer indicated clips not watched in full	32	20	36	105
***Demographics***				
Gender:				
Female (%)	68.7	79.4	68.8	53.4
Male (%)	30.8	20.6	30.8	45.8
Identified another way (%)	.5	-	-	.8
Not reported (%)	-	-	.4	-
Age:				
Range	18–78	18–37	18–85	19–80
Mean	36.83	27.84	27.52	37.74
Standard Deviation	12.29	4.64	11.73	11.76

^1^Attention checks were as follows: “Please select ‘disagree’ to show you are paying attention, and “Please select ‘5’ to demonstrate that you are reading this survey carefully”.

^2^ Participants that responded “no” to the following question were excluded: “Please answer honestly, did you watch this entire video from start to finish without any interruptions?”

### Measures

#### Positive and negative affect

The Positive and Negative Affect Schedule (PANAS; [61]) was administered both before participants viewed the clip(s) and again immediately after. The PANAS consists of 20 adjectives comprising positive and negative affect subscales. Participants used a 5-point scale from 1 (*very slightly or not at all*) to 5 (*extremely*) to indicate their responses. Before the video clip, participants completed the items in reference to how they “currently” felt. After the video clip, participants completed the items in reference to how they “currently” felt (Study 1a), how they felt “whilst watching the video clip” (Study 1b), or how they felt “right now, after what you have just seen” (Studies 1c-d). These different points of time-reference in the instructions were unintentional, but as a) our main variable of interest was change in affect (and the same time-reference was used both pre- and post-media exposure) and b) the reliability of the PANAS is unaffected by the time instructions that are used [61], this inconsistency should not affect our results. Alpha coefficients were good-excellent across all studies for both time points (all α’s >.84).

#### Elevation

Elevation was measured using six items representing the physical sensations, and positive thoughts and feelings that represent the experience of elevation [30]. Specifically, participants rated the extent to which they experienced feeling ‘moved’, ‘uplifted’, ‘optimistic about humanity’, that they had ‘a warm feeling in the chest’, and wanted ‘to help others’ and ‘become a better person’ using a 9-point scale from 1 (*not at all*) to 9 (*very strongly*). Participants provided responses in reference to each item “currently” (Study 1a), “while watching the video clip” (Study 1b), and “right now” (Studies 1c-d). The scale had good-excellent reliability across all studies (all α’s >.84). Additional items were embedded within the elevation scale (Specifically, the following items were also administered. Study 1a: awe, admiration, lump in throat. Study 1c: happy. Study 1d: admiration, respect, gratitude, love.) but, for consistency of measurement, only the items that were included in all studies have been analysed.

#### Amusement (Study 1d only)

Participants indicated the extent to which they were feeling “entertained” and “amused” using a 9-point scale from 1 (*Not at all*) to 9 (*Very strongly*). Previous research indicates that these two feelings are frequent responses to humorous stimuli [28]. These items were embedded within the elevation scale and were used to check the validity of the amusement stimuli included in Study 1d.

#### Perceptions of humanity

In Studies 1a, 1b, and 1d (A researcher error meant that this scale was not assessed in Study 1c.) we measured perceptions of humanity using a 3-item (As per the original subscale we measured all four items in the social well-being subscale. However of the four items, one item focused participants’ attention on the extent to which they felt they had something important to contribute to society, while the other three items asked participants to indicate how positively they felt about people and society. Given this difference in focus, and the fact that removing the item improved the reliability of the measure in Studies 1a and 1b, we did not include it in any of our analyses.) scale adapted from the social well-being facet of the Mental Health Continuum Short-Form [62]. The three chosen items reflected social acceptance, i.e., having favourable views of human nature (“People are basically good”), social coherence, i.e., having a sense of understanding of the world (“The way our society works makes sense to you”), and social actualisation, i.e., believing societal potential is being fulfilled (“Our society is a good place, or is becoming a better place for all people”). After viewing the video clip, participants indicated the extent to which they agreed/disagreed with each item using a 7-point scale from 1 (*strongly disagree*) to 7 (*strongly agree*). The scale had acceptable-good reliability across all studies (all α’s >.72).

#### Qualitative responses

Participants were given the opportunity to provide further feedback on their personal reaction to the stimuli. Specifically we provided an empty text box and asked them “What thoughts and feelings did you experience while watching the video(s)?”

## Results

### Open data practises

All data and associated syntax for Studies 1a-d and Study 2 are available at: https://osf.io/mw79n.

### Change in affect after watching the news stories

**Fig 1:**
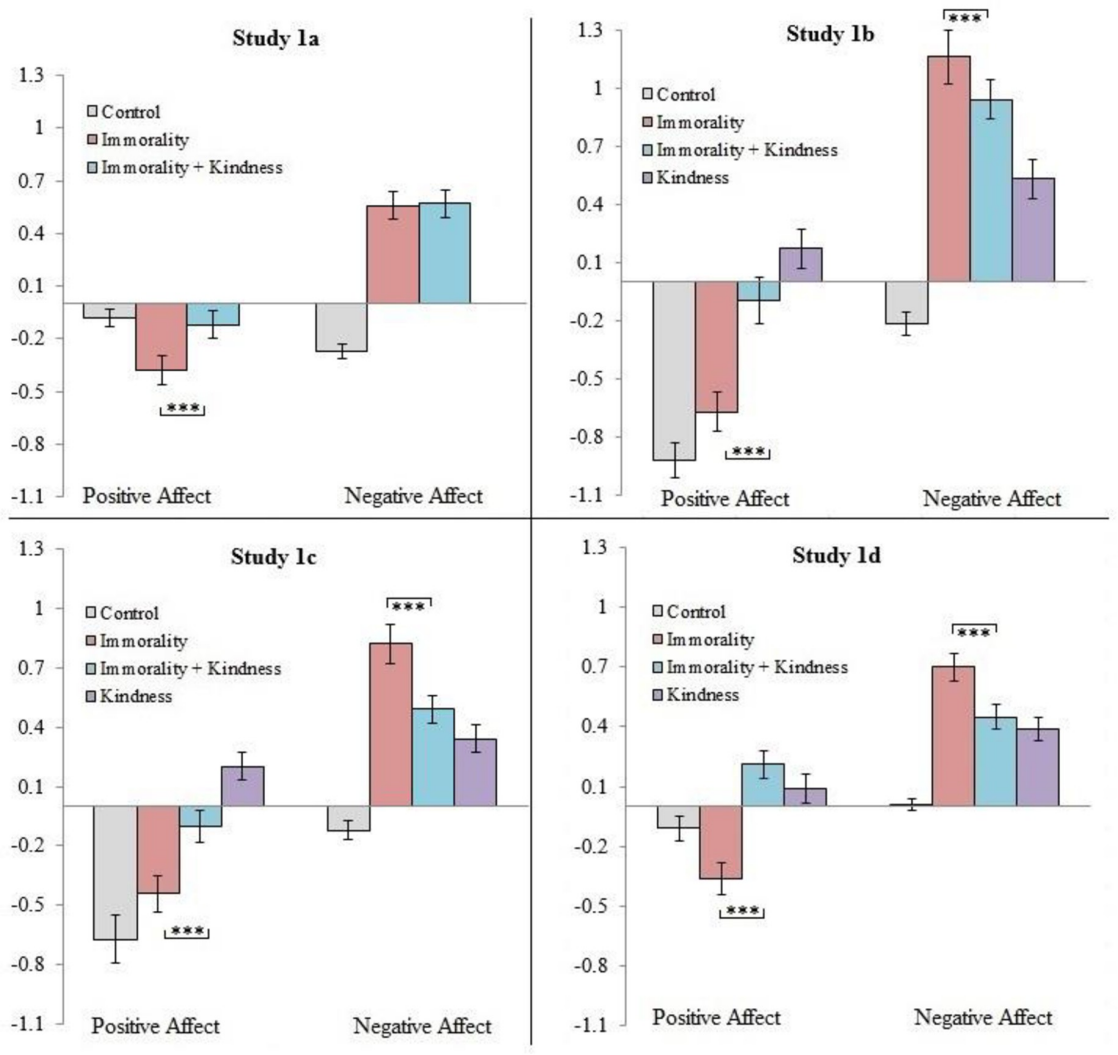
Studies 1a-d: Differences in the Immorality vs. Immorality+Kindness conditions in changes to affective well-being. *Note*. Shown are the results from post-hoc pairwise t-tests depicting the significance of differences for the comparisons between the Immorality and Immorality+Kindness conditions. Numbers below 0 indicate a decrease in affect; numbers above 0 indicate an increase in affect. Error bars represent standard errors. *** *p* < .001.

In order to better understand whether pairing footage of others’ immorality with footage of others’ kindness would alleviate some of the negative well-being impacts of the immorality, we first examined the pre-post changes to affect prompted within each condition by watching either the Immorality, Immorality+Kindness, Kindness or Control video clips. Accordingly, we conducted paired sample t-tests for each condition within each study. We then used a within subjects effect size calculator to obtain Cohen’s d (see https://camel.psyc.vt.edu/models/stats/effect_size.shtml) before using version 2 of Goh’s mini meta-analysis Excel template (see https://osf.io/8yubf) to convert our effect sizes from Cohen’s *d* to Pearson’s *r* (equal n), and to conduct a mini-meta analysis across Studies 1a-d [63]. The results are displayed in Table 3, and changes in affect are illustrated in Fig 1.

**Table 3:** Studies 1a-d: Means and standard deviations for positive and negative affect, and paired sample t-test results.

Condition	Affect	Study	Time 1		Time 2		*Mean Difference*	Paired Sample T-tests						Meta-Analysis
			Mean	*SD*	Mean	*SD*		*t*	*SE*	*p*	95% CI	*d*	*r*	
Control	Positive	1a	2.95	.90	2.87	.99	-.08	-1.54	.05	.128	[-.18, .02]	.18	-.09	***r* = -.17, SE = .07, CI95 = [-.29, -.04], Z = -2.58, *p* = .010**
		1b	**2.55**	**.80**	**1.63**	**.52**	**-.92**	**-9.82**	**.09**	**< .001**	**[-1.11, -.73]**	**-.43**	**-.21**	
		1c	**2.31**	**.81**	**1.64**	**.65**	**-.67**	**-5.70**	**.12**	**< .001**	**[-.91, -.43]**	**-.92**	**-.42**	
		1d	2.91	.99	2.80	1.04	-.11	-1.79	.06	.076	[-.22, .01]	-.20	-.10	
	Negative	1a	**1.55**	**.65**	**1.28**	**.54**	**-.27**	**-5.72**	**.05**	**< .001**	**[-.36, -.17]**	**-.69**	**-.33**	***r* = -.17, SE = .07, CI95 = [-.29, -.04], Z = -2.62, *p* = .009**
		1b	**1.45**	**.71**	**1.23**	**.56**	**-.22**	**-3.38**	**.06**	**.002**	**[-.34, -.09]**	**-.52**	**-.25**	
		1c	**1.49**	**.63**	**1.37**	**.51**	**-.12**	**-2.23**	**.05**	**.032**	**[-.23, -.01]**	**-.37**	**-.18**	
		1d	1.31	.65	1.31	.61	-.001	-0.03	.03	.973	[-.06, .06]	-.001	-.001	
Immorality	Positive	1a	**2.91**	**.93**	**2.54**	**.88**	**-.38**	**-4.96**	**.08**	**< .001**	**[-.53, -.23]**	**-.56**	**-.27**	***r* = -.33, SE = .06, CI95 = [-.44, -.21], Z = -5.28, *p* < .001**
		1b	**2.59**	**1.03**	**1.91**	**.53**	**-.67**	**-6.35**	**.11**	**< .001**	**[-.89, -.46]**	**-.93**	**-.42**	
		1c	**2.61**	**.78**	**2.18**	**.61**	**-.44**	**-5.05**	**.09**	**< .001**	**[-.61, -.26]**	**-.81**	**-.38**	
		1d	**2.82**	**.76**	**2.46**	**.76**	**-.36**	**-6.10**	**.06**	**< .001**	**[-.47, -.24]**	**-.66**	**-.31**	
	Negative	1a	**1.59**	**.75**	**2.15**	**.90**	**.56**	**6.92**	**.08**	**< .001**	**[.40, .72]**	**.80**	**.37**	***r* = .45, SE = .06, CI95 = [.35, .55], Z = 7.54, *p* < .001**
		1b	**1.32**	**.47**	**2.48**	**.87**	**1.16**	**8.17**	**.14**	**< .001**	**[.87, 1.44]**	**1.20**	**.51**	
		1c	**1.39**	**.41**	**2.21**	**.77**	**.82**	**8.43**	**.10**	**< .001**	**[.62, 1.01]**	**1.42**	**.58**	
		1d	**1.35**	**.63**	**2.05**	**.76**	**.70**	**9.29**	**.07**	**< .001**	**[.55, .85]**	**.98**	**.44**	
Immorality +Kindness	Positive	1a	2.94	.77	2.82	.84	-.12	-1.50	.08	.139	[-.28, .04]	-.18	-.09	*r* = .01, SE = .04, CI95 = [-.08, .10], Z = .28, *p* = .779
		1b	2.74	.87	2.65	.75	-.10	-0.78	.12	.440	[-.34, .15]	-.11	-.05	
		1c	2.48	.90	2.38	.77	-.10	-1.21	.08	.230	[-.26, .06]	-.14	-.07	
		1d	**2.89**	**.96**	**3.10**	**.97**	**.21**	**3.21**	**.06**	**.002**	**[.08, .34]**	**.38**	**.19**	
	Negative	1a	**1.56**	**.69**	**2.13**	**.80**	**.57**	**7.09**	**.08**	**< .001**	**[.41, .74]**	**.87**	**.40**	***r* = .40, SE = .06, CI95 = [.29, .50], Z = 6.64 *p* < .001**
		1b	**1.38**	**.42**	**2.32**	**.71**	**.94**	**9.42**	**.10**	**< .001**	**[.74, 1.14]**	**1.41**	**.58**	
		1c	**1.51**	**.65**	**2.00**	**.69**	**.49**	**6.99**	**.07**	**< .001**	**[.35, .64]**	**.80**	**.37**	
		1d	**1.32**	**.62**	**1.77**	**.71**	**.45**	**5.56**	**.08**	**< .001**	**[.29, .61]**	**.66**	**.31**	
Kindness	Positive	1b	2.56	.89	2.72	.78	.17	1.65	.10	.106	[-.04, .37]	.24	.12	*r* = .11, SE = .07, CI95 = [-.02, .24], Z = 1.65, *p* = .099
		1c	**2.44**	**.84**	**2.64**	**.80**	**.20**	**2.67**	**.07**	**.009**	**[.05, .34]**	**.29**	**.14**	
		1d	2.77	.88	2.87	.96	.09	1.37	.07	.173	[-.04, .23]	.16	.08	
	Negative	1b	**1.41**	**.58**	**1.94**	**.69**	**.53**	**5.30**	**.10**	**< .001**	**[.33, .73]**	**.80**	**.37**	***r* = .31, SE = .07, CI95 = [.18, .43], Z = 4.64, *p* < .001**
		1c	**1.52**	**.53**	**1.87**	**.70**	**.34**	**5.05**	**.07**	**< .001**	**[.21, .48]**	**.54**	**.26**	
		1d	**1.29**	**.52**	**1.69**	**.70**	**.39**	**6.43**	**.06**	**< .001**	**[.27, .52]**	**.71**	**.33**	
Condition	Affect		*Time 1*		*Time 2*		*Mean Difference*	Paired Sample T-tests						
		Study	Mean	*SD*	Mean	*SD*		*t*	*SE*	*p*	95% CI	*d*	*r*	
Amusement	Positive	1d	2.84	.96	2.85	1.01	.01	0.31	.05	.756	[-.08, .11]	.02	-	-
	Negative	1d	**1.38**	**.67**	**1.27**	**.55**	**-.12**	**-2.72**	**.04**	**.008**	**[-.21, -.04]**	**.26**	-	-
Immorality+ Amusement	Positive	1d	**3.07**	**.94**	**2.90**	**.91**	**-.17**	**-2.69**	**.06**	**.009**	**[-.29, -.04]**	**.30**	-	-
	Negative	1d	**1.40**	**.68**	**1.68**	**.76**	**.28**	**3.61**	**.08**	**.001**	**[.13, .44]**	**.40**	-	-

*SD* = Standard Deviation. SE = Standard Error. CI = Confidence Intervals. Bold denotes a significant effect. Within subject effect sizes were calculated using https://camel.psyc.vt.edu/models/stats/effect_size.shtml which applies the formula used by G*Power

As expected, viewing the Immorality clip had an adverse impact on participants’ emotional well-being, as participants reported significant increases in negative affect and significant decreases in positive affect (respectively, meta-analytic *r* = .45, *r* = -.33, both *p*’s < .001). In contrast, participants in the Immorality+Kindness condition appeared less adversely affected; while they reported increases in negative affect (*r* = .40, *p* < .001), their positive affect remained unaffected (meta-analytic *r* = .01, *NS*). Taken together, these results suggest that exposure to others’ acts of kindness following exposure to others’ immorality appears to offset some of the adverse emotional impacts of viewing others’ immorality, at least for positive affect.

Surprisingly, the Control condition, which was intended to be affectively neutral, was instead affectively mixed as it significantly reduced both positive affect and negative affect (respectively, both *r’s* = -.17, both *p*’s < .001). The qualitative feedback from participants suggests these mixed affective responses were due to finding the clips boring and irrelevant. This attests to the unpleasant experience of boredom inherent in its definition as “the aversive experience of wanting, but being unable to engage in satisfying activity” [61, 64 p.482].

### Elevation and perceptions of humanity after watching the news stories

To examine the effect of condition on elevation and perceptions of humanity we conducted two ANOVAs, one per dependent variable. There was a significant effect of condition on elevation for Studies 1a-d (Study 1a: *F*(2, 208) = 23.98, *p* < .001, η^2^ = .19; Study 1b: *F*(3, 176) = 87.04, *p* < .001, η^2^ = .60. Study 1c: *F*(3, 236) = 75.47, *p* < .001, η^2^ = .49; Study 1d: *F*(5, 499) = 50.89, *p* < .001, η^2^ = .34). There was also a significant effect of condition on perceptions of humanity in Studies 1a and 1b (Study 1a: *F*(2, 208) = 4.02, *p* = .019, η^2^ = .04; Study 1b: *F*(3, 176) = 8.24, *p* < .001, η^2^ = .12), but not Study 1d (*F*(5, 499) = .98, *NS*, η^2^ = .01). Table 4 shows the means and standard deviations for both elevation and perceptions of humanity in each condition. The highest levels of elevation and the most positive perceptions of humanity were reported by participants in either the Kindness or Immorality+Kindness conditions.

**Table 4:** Studies 1a-d: Means and standard deviations for elevation and perceptions of humanity.

Study	Condition	Elevation		Perceptions of humanity	
		Mean	*SD*	Mean	*SD*
1a	Control	4.74	1.72	3.94	1.22
	Immorality	4.67	1.46	3.65	1.26
	Immorality+Kindness	6.35	1.62	4.24	1.20
1b	Control	1.86	1.38	4.62	1.34
	Immorality	3.77	1.27	3.58	1.30
	Immorality+Kindness	6.08	1.74	4.50	1.16
	Kindness	6.49	1.72	4.70	1.11
1c	Control	1.69	1.07	-	-
	Immorality	3.65	1.13	-	-
	Immorality+Kindness	5.38	1.68	-	-
	Kindness	6.18	1.96	-	-
1d	Control	2.29	2.07	4.61	1.34
	Immorality	3.72	1.73	4.42	1.05
	Immorality+Kindness	6.68	1.82	4.81	1.27
	Kindness	6.33	2.35	4.58	1.21
	Immorality+Amusement	4.82	2.11	4.57	1.01
	Amusement	4.61	2.36	4.47	1.26

SD = Standard Deviation

### Immorality vs. Immorality+Kindness: Can kindness alleviate the aversive effects of seeing others’ immorality?

We next carried out planned contrasts comparing the Immorality condition to the Immorality+Kindness condition to see if kindness could alleviate the aversive impacts of exposure to immorality. We then converted the resulting effects sizes from Cohen’s d to Pearson’s r (using the unequal *n* formula) and conducted a mini-meta analysis across Studies 1a-d [63, see also https://osf.io/8yubf].

As expected, participants in the Immorality+Kindness condition fared significantly better than participants in the Immorality condition (see Table 5 and Fig 1). Specifically, participants in the Immorality+Kindness condition reported significantly lower reductions to positive affect, significantly lower increases to negative affect, and significantly more positive perceptions of humanity than participants in the Immorality condition. Therefore, the data provide support for the idea that witnessing others’ kindness can alleviate the adverse impacts prompted through media exposure to an act of immorality.

**Table 5:** Studies 1a-d: Examining the effects of the immorality condition vs. The Immorality+Kindness condition on changes to affect, elevation, and perceptions of humanity.

Outcome Variable	Study	*Immorality+Kindness*		*Immorality*		Planned Contrasts							Meta-analysis
		Mean	*SD*	Mean	*SD*	*Mean Difference*	*SE*	*t*	*p*	95% CI	*d*	*r*	
Change in PA	**1a**	**-.12**	**.64**	**-.38**	**.66**	**.26**	**.10**	**2.60**	**.010**	**[.06, .46]**	**.40**	**.20**	***r* = .33, SE = .04, CI95 = [.24, .40]. *Z* = 7.52, *p* < .001**
	**1b**	**-.10**	**.82**	**-.67**	**.73**	**.58**	**.15**	**3.89**	**< .001**	**[.28, .87]**	**.73**	**.34**	
	**1c**	**-.10**	**.71**	**-.44**	**.53**	**.34**	**.14**	**2.46**	**.015**	**[.07, .61]**	**.55**	**.26**	
	**1d**	**.21**	**.55**	**-.36**	**.55**	**.57**	**.09**	**6.46**	**< .001**	**[.39, .74]**	**1.04**	**.46**	
Change in NA	1a	.57	.65	.56	.70	.01	.11	0.16	.874	[-.18, .22]	.01	.01	***r* = .14, SE = .04**, **CI95 = [.05, .22]. *Z* = 3.05, *p =* .002**
	1b	.94	.67	1.16	.97	-.22	.15	-1.45	.149	[-.51, .08]	-.26	.13	
	**1c**	**.49**	**.61**	**.82**	**.58**	**-.32**	**.12**	**-2.65**	**.010**	**[-.56, -.08]**	**-.53**	**.24**	
	**1d**	**.45**	**.68**	**.70**	**.71**	**-.25**	**.11**	**-2.29**	**.023**	**[-.47, -.04]**	**-.37**	**.18**	
Elevation	**1a**	**6.35**	**1.62**	**4.67**	**1.46**	**1.69**	**.27**	**6.24**	**< .001**	**[1.15, 2.22]**	**1.09**	**.48**	***r* = .56, SE = .04**, **CI95 = [.49, .61]. *Z* = 13.92, *p* < .001**
	**1b**	**6.08**	**1.74**	**3.77**	**1.27**	**2.31**	**.32**	**7.29**	**< .001**	**[1.69, 2.94]**	**1.53**	**.61**	
	**1c**	**5.38**	**1.68**	**3.65**	**1.13**	**1.73**	**.27**	**6.41**	**< .001**	**[1.20, 2.27]**	**1.11**	**.46**	
	**1d**	**6.68**	**1.82**	**3.72**	**1.73**	**2.96**	**.28**	**10.50**	**< .001**	**[2.40, 3.52]**	**1.67**	**.64**	
Perceptions of Humanity	**1a**	**4.24**	**1.20**	**3.65**	**1.26**	**.59**	**.21**	**2.84**	**.005**	**[.18, 1.00]**	**.48**	**.23**	***r* = .23, SE = .05**, **CI95 = [.14, .21]. *Z* = 4.69, *p* < .001**
	**1b**	**4.50**	**1.16**	**3.58**	**1.30**	**.91**	**.26**	**3.55**	**< .001**	**[.41, 1.42]**	**.75**	**.35**	
	**1d**	**4.81**	**1.27**	**4.42**	**1.05**	**.39**	**.19**	**2.08**	**.040**	**[.02, .76]**	**.34**	**.17**	

*Note*: *SD* = Standard Deviation. SE = Standard Error. CI = Confidence Intervals. Bold denotes a significant effect.

### Are light-hearted news stories as effective as kindness news stories?

In Study 1d, the inclusion of Amusement and Immorality+Amusement conditions allowed us to compare whether pairing immorality with kindness was more effective in alleviating the negative impacts of seeing others’ immorality than pairing immorality with a light-hearted news story intended to provoke amusement. We first confirmed that amusement varied between the conditions by conducting a one-way ANOVA which showed condition had the desired effect on amusement, *F*(5, 499) = 35.15, *p* < .001, η^2^ = .26. Using follow-up planned contrasts we confirmed that the Amusement condition was rated as significantly more amusing than the other conditions (see Table 6).

**Table 6:** Study 1d: Means and standard deviations for amusement per condition, and planned comparisons.

Condition	Mean	*SD*	Comparison to Amusement Condition
Amusement	5.74	2.26	-
Control	3.55	2.36	*t*(175) = 6.31, *p* < .001, CI95 = [1.50,2.87]
Immorality	1.60	1.29	*t*(175) = 14.97, *p* < .001, CI95 = [3.60,4.69]
Immorality+Kindness	2.92	2.23	*t*(158) = 7.90, *p* < .001, CI95 = [2.11,3.52]
Kindness	2.88	2.19	*t*(171) = 8.46, *p* < .001, CI95 = [2.19,3.53]
Immorality+Amusement	3.65	2.44	*t*(168) = 5.76, *p* < .001, CI95 = [1.37,2.20]

*SD* = Standard Deviation. CI = Confidence Interval.

Exposure to amusing clips helped to alleviate some of the aversive effects of exposure to the immorality clips. Specifically, relative to participants in the Immorality condition, participants in the Immorality+Amusement condition experienced significantly smaller reductions to positive affect, smaller increases in negative affect and greater levels of elevation but did not differ on perceptions of humanity (see Table 7). This indicates that pairing the act of immorality with an amusing video helped alleviate the aversive emotional effects of seeing others’ immorality, but not the effects on perceptions of humanity.

**Table 7:** Study 1d: Relative effectiveness of Immorality+Kindness and Immorality+Amusement vs. Immorality in their impacts on changes to affective well-being, elevation and perceptions of humanity.

	Planned comparison: Immorality vs. Immorality+Kindness						Planned comparison: Immorality vs. Immorality+Amusement					
	*Mean Difference*	*SE*	*t*	*p*	95% CI	*d*	*Mean Difference*	*SE*	*t*	*p*	95% CI	*d*
Change in PA	**.57**	**.09**	**6.46**	**< .001**	**[.39, .74]**	**1.04**	**.19**	**.09**	**2.21**	**.028**	**[.02, .36]**	**.34**
Change in NA	**-.25**	**.11**	**-2.29**	**.023**	**[-.47, -.04]**	**-.37**	**-.42**	**.11**	**-3.87**	**< .001**	**[-.63, -.21]**	**-.60**
Elevation	**2.96**	**.28**	**10.55**	**< .001**	**[2.40, 3.52]**	**1.67**	**1.10**	**.30**	**3.71**	**< .001**	**[.51, 1.68]**	**.57**
Humanity	**.39**	**.19**	**2.08**	**.040**	**[.02, .76]**	.34	.15	.16	0.95	.343	[-.16, .46]	.14

Note. PA = Positive Affect, NA = Negative Affect, SE = Standard Error. CI = Confidence Intervals. Bold denotes a significant effect.

For three out of our four outcome measures, amusement appeared to be less effective than kindness in mitigating the negative effects of the immorality; Immorality+Kindness had a larger effect than Immorality+Amusement on combatting reductions to positive affect (*d* = 1.04 vs. *d* = .34), and prompted greater experiences of elevation (*d* = 1.67 vs. *d* = .57) and more positive perceptions of humanity (*d* = .34 vs. *d* = .14; see Table 7).

## Discussion

Across four samples, we replicated previous findings showing that exposure to media coverage of terrorism can have an immediate and aversive impact on affective well-being and perceptions of humanity [6, 12]. Importantly, for the first time, we found that these negative effects could be alleviated through media exposure to the acts of kindness that occurred in the aftermath of terrorism. Specifically, we found that participants in the Immorality+Kindness condition had a less emotionally aversive experience than participants in the Immorality only condition, as they experienced a lower drop in positive emotions, and a lower increase in negative emotions, greater elevation and more positive perceptions of humanity. Moreover, amusement was not as effective in alleviating the negative effects of exposure to others’ immorality as seeing others’ kindness. Such findings suggest that there is something uniquely powerful and restorative about seeing others’ kindness that is not simply attributable to it triggering pleasant feelings.

While witnessing others’ kindness had several benefits compared to seeing news featuring immorality alone, it still resulted in significant increases to negative affect. We suspect this may partly have been because, while the kindness clip did not show footage of the terrorism, it still reminded people that the terrorist act had happened. As such, it is not possible to separate the kindness and the positive emotions it evoked from the negative emotional backdrop of the situation. Indeed, this may have been the reason that, in our previous research, reading tweets about kindness in response to the COVID-pandemic did not have the predicted positive effect on mood relative to a no-information control group [10]. To examine this further, in Study 2 we ensured the acts of kindness were completely unrelated to the acts of others’ immorality to avoid unwittingly reiterating exposure to others’ immorality within the kindness condition.

## Study 2

In Study 2, we tested the generalizability of our findings by changing five study features. First, we used a more diverse range of immoral and kind stimuli (e.g., not focusing solely on terrorism). We also changed the ratio of Immoral to Kindness news stories, so that participants saw three news stories featuring kind acts, rather than five. Second, we presented the stimuli as news stories to be read, rather than video clips to be watched, to see if the findings would replicate despite the use of a different, and potentially less evocative news medium. Third, we ensured that the content of the Kindness news stories was completely unrelated to the content of the news stories featuring immorality. Fourth, we utilised a No-Treatment Control condition, since the media clips that we used as our control condition in Studies 1a-d were not judged to be as neutral as we had hoped, rendering them ineffective as a point of comparison. Fifth, we tested the idea that seeing others’ kindness would provide wider societal benefits in the form of an increased willingness to act pro-socially. Indeed, a recent meta-analysis of more than 25,000 people finds that when people see someone model prosocial behaviour, they tend to act more pro-socially themselves [65].

## Method

### Ethics approval

Prior to commencement of this research, Study 2 was reviewed by the Faculty of Science and Health Ethics Committee at the University of Essex and granted approval with the following code: ETH2122-0166. Prior to participation in the online study, participants provided “written” consent by selecting the response “I agree to give my consent to participate in this research”.

### Participants

An a priori power analysis in G*Power indicated that a sample of 660 participants (110 in each of the six conditions) was required to detect a small-to-medium effect (*f* = .14) with 80% power and a one-tailed alpha of .05. Participants were recruited via Prolific Academic until the target sample size was reached. Data were excluded from participants that had not completed the survey in full (*n* = 5) or who did not pass the attention checks (*n* = 2). Further data was collected beyond the sample size (*n* = 5) to achieve more evenly balanced sample sizes per condition. The final sample consisted of 665 participants (aged 18–76, *M*_*age*_ = 32.64, *SD* = 11.61; 73% female; 86% White.

### Study design

Participants were randomly allocated to one of six conditions: No-Treatment Control (*n* = 113); Immorality (*n* = 115); Immorality+Kindness (*n* = 115); Kindness (*n* = 106); Amusement (*n* = 112); Immorality+Amusement (*n* = 104). Participants viewed news content related to their condition (e.g., participants in the Immorality condition saw a news story featuring immorality, while participants in the Immorality+Kindness condition saw one news story featuring immorality, followed by three news stories featuring kindness). Further details about the content of the stimuli are provided below.

### Procedure

Before reading the news story/stories, participants completed the PANAS [61]. Participants then read various news content depending on which condition they were randomly allocated to (or no news content, in the No-Treatment Control condition). To encourage participants to read the content shown we did not allow them to proceed to the next part of the survey until 20 seconds had passed. Immediately after reading the news story/stories participants in all conditions except the No-Treatment Control condition completed the PANAS again, followed by measures of elevation and amusement, and perceptions about the goodness of others [63]. After responding to the first PANAS [61] measure, participants in the No-Treatment Control condition, were simply informed that they would be asked further questions about their mood, feelings and beliefs about others. They were then asked to complete the measures of elevation and amusement, before responding a second time to the PANAS measure. Finally, participants in all conditions completed the dictator game as a proxy of their own pro-sociality [66] before responding to exploratory questions surrounding their perceptions of others’ kind acts (not reported; see OSF for full materials).

### Stimuli

Seven different news stories were collated to represent each category (Immorality, Kindness, and Amusement). In the Immorality condition, participants were allocated to view one out of seven possible news stories. In the Kindness and Amusement conditions, participants viewed three out of seven possible news stories. In the Immorality+Kindness and Immorality+Amusement participants viewed a total of four news stories (one immoral, out of a possible seven options, and three kind/amusing out of a possible seven options).

**Fig 2:**
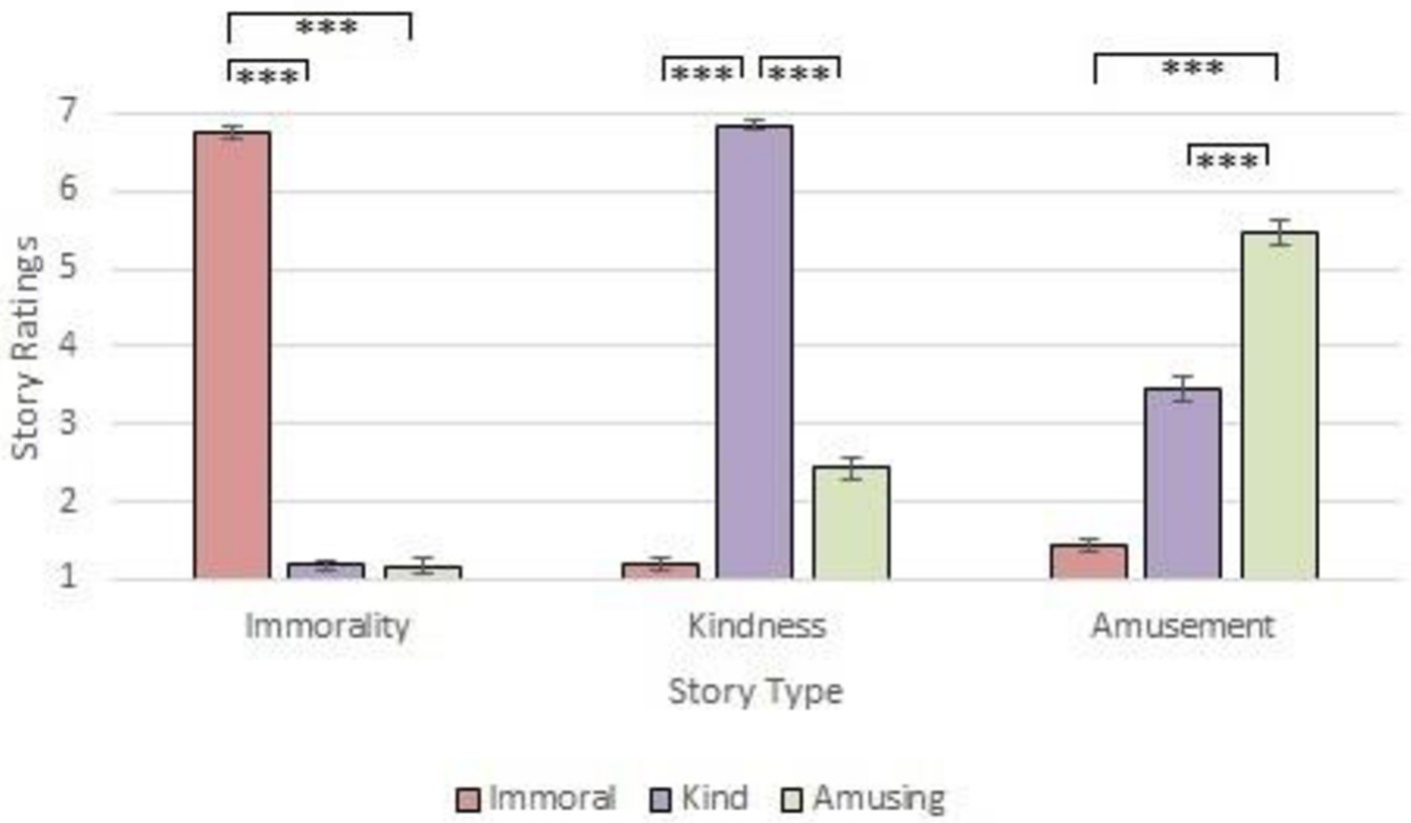
Study 2: Establishing content validity of news stories. *Note*. Shown are the results from post-hoc pairwise t-tests depicting the content validity of immoral, kind and amusing news stories in Study 2.. Error bars represent standard errors. *** *p* < .001.

Prior to the study, all stimuli were piloted to ensure they were appropriate for their designated condition (see analyses on OSF). As shown in Fig 2, the news stories selected scored highly in the domain they were designed to represent (e.g., the stimuli in the kindness condition were judged to be kinder than the stimuli in the immoral and amusing conditions). A summary of the content included in each news story is displayed in Table 8, and the stimuli are available in full via OSF (https://osf.io/mw79n).

**Table 8:** Study 2: Stimuli content per condition.

Condition	News	Summary
Immorality	Puppy Killer	A man kicked and killed his girlfriend’s puppy.
	Sex Offender	Repeat sex offender found in possession of child rape images.
	Evil Doctor	Oncologist falsely diagnosed and treated patients for health insurance money.
	Brutal Murder	Man found guilty of brutally murdering his partner.
	Glass Attack	Teenager seriously injured after being attacked with a bottle by an unknown stranger.
	Bullying Brag	Bullies share footage of themselves attacking a teenager at a playground on social media.
	COVID-19 Scam	Huge surge in con artists impersonating tax officials to gain financial details during COVID-19.
Kindness	Free Ambulance Service	Man in India runs his own free makeshift motorcycle powered ambulance to get those in rural locations to hospital.
	Birthday Money Surrender	9-yr old uses birthday money to buy food for the homeless.
	Instagram Hero	Woman intervenes to help suicidal Instagram users
	Car Gift for Co-Worker	Successful fund raising initiative to buy a co-worker a car so she no longer has to walk 12 miles to work.
	Haircuts for Homeless	Hairdresser offers free haircuts for the homeless in her city.
	Vet for Strays	Vet seeks out and treats homeless people’s pets for free.
	Generous Tip	Hair Stylist surprised with $25000 tip.
Amusement	Rick Rolling	Student hides words to Rick Astley’s “Never Gonna Give You Up” in his quantum physics essay.
	Rude Street Names	10 rude street names in South England.
	Swearing Parrots	Wildlife centre removed cheeky swearing parrots out of public’s earshot.
	Bath Puff Fascinator	Woman wears bath time aid as accessory to a wedding.
	Tourist Trap	American Tourist locked in British book store is rescued after tweeting for help.
	Joke Award	Following joke deemed award worthy: “I needed a password eight characters long so I picked Snow White and the Seven Dwarves”
	Hard to Say	Most difficult to pronounce words revealed

### Measures

#### Positive and negative affect

As in Studies 1a-d, the PANAS [61] was administered both before participants viewed the news story/stories and again after. Participants completed the items about how they felt “right now” (control condition) or “right now, after reading that news story/those news stories” (experimental conditions). Alpha coefficients were good-excellent for both time points (all α’s >.88).

#### Elevation and amusement

Participants indicated the extent to which they were experiencing 6 items assessing elevation (as per Studies 1a-d and [30]), and 3 items assessing amusement (as per Study 1d and [28] but with the inclusion of the additional item “like laughing”). Both the elevation and amusement scales had good-excellent reliability (respectively, α = .88 and .90).

#### Perceptions of humanity

Participants indicated their agreement/disagreement with 8 items measuring their belief in the benevolence of people (e.g., “People are basically kind and helpful”) and the impersonal world (e.g., “The world is a good place”) [67]. Participants provided their responses using a 6-point scale ranging from 1 (*Strongly disagree*) to 6 (*Strongly Agree*). The scale had good reliability (α = .86).

#### Prosociality

Participants were asked to anonymously allocate a hypothetical £100 between themselves and an unknown recipient. Past studies have argued this constitutes a proxy for pro-sociality as participants have the opportunity to behave kindly by allocating larger sums to the stranger or to behave selfishly by allocating larger sums to themselves without any consequences [66].

## Results

### Changes in affect after reading the news stories

**Fig 3:**
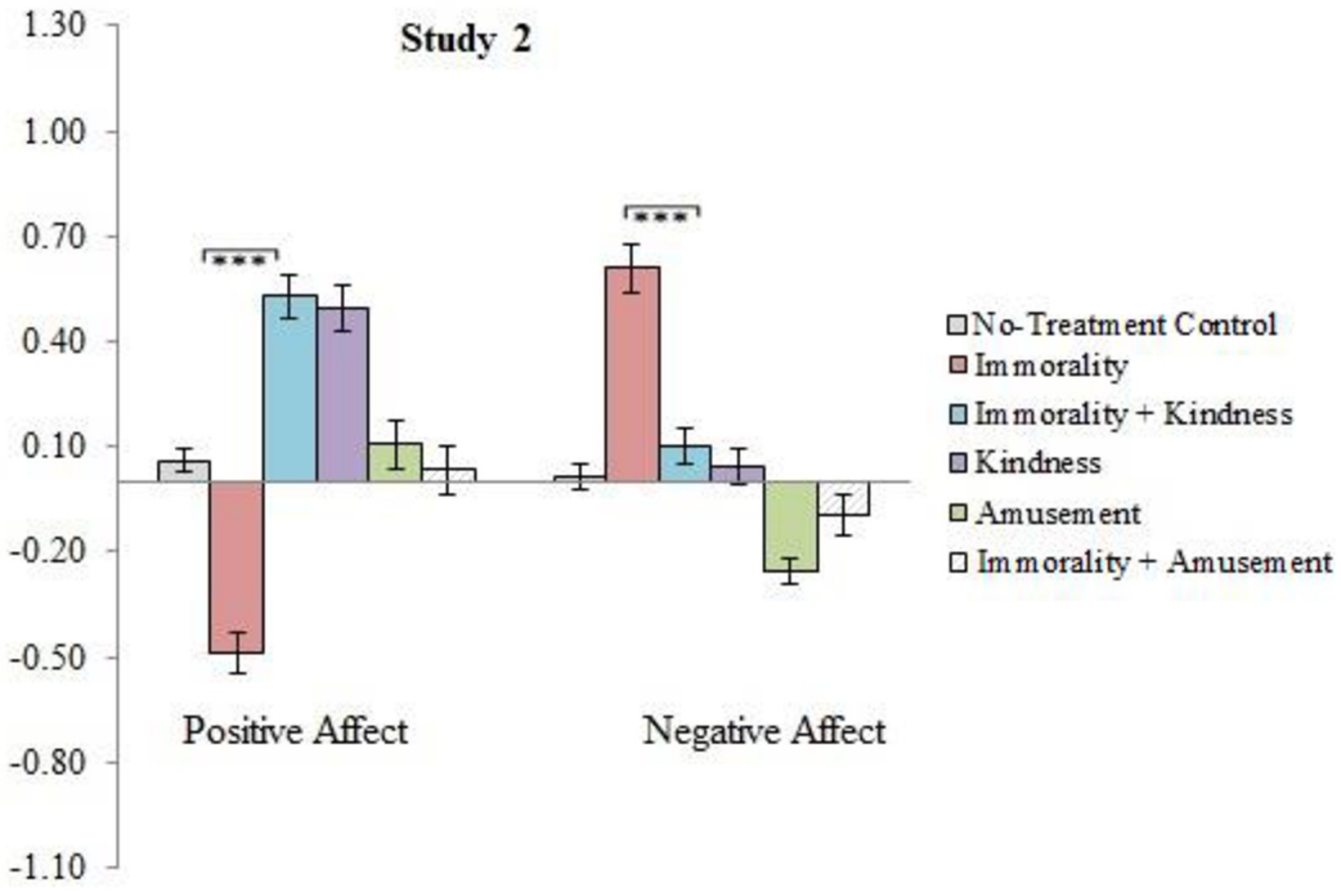
Study 2: Differences in the Immorality vs. Immorality+Kindness conditions in changes to affective well-being. *Note*. Shown are the results from post-hoc pairwise t-tests depicting the significance of differences for the comparisons between the Immorality and Immorality+Kindness conditions. Numbers below 0 indicate a decrease in affect; numbers above 0 indicate an increase in affect. Error bars represent standard errors. *** *p* < .001.

To understand the impact of condition on changes to positive and negative affect, we conducted paired sample t-tests for each condition. The results are displayed in Table 9, and changes in affect are illustrated in Fig 3. Reading a news story featuring immorality led to significant decreases in positive affect and significant increases in negative affect. In contrast, when the news story featuring immorality was followed by three news stories featuring acts of kindness, while participants still reported small, albeit significant increases to negative affect, they also experienced significant increases in positive affect. Following the immorality story with three amusing news stories did not prompt significant changes in either positive or negative affect.

**Table 9:** Study 2: Means and standard deviations for positive and negative affect, and paired sample t-test results.

Affect	Condition	*n*	Before News Stories		After News Stories							
			Mean	*SD*	Mean	*SD*	Mean Difference	*t*	*SE*	*p*	95% CI	*d*
Positive	No-Treatment Control	113	2.69	.77	2.76	.98	.06	1.73	.03	.086	[-.01, .13]	.19
	**Immorality**	**115**	**2.54**	**.76**	**2.05**	**.71**	**-.49**	**-8.22**	**.06**	**< .001**	**[-.61, -.37]**	**.76**
	**Immorality + Kindness**	**115**	**2.66**	**.76**	**3.19**	**.97**	**.53**	**8.40**	**.06**	**< .001**	**[.41,.65]**	**.74**
	**Kindness**	**106**	**2.71**	**.75**	**3.21**	**1.02**	**.49**	**7.89**	**.06**	**< .001**	**[.37,.62]**	**.78**
	Amusement	112	2.88	.71	2.99	.99	.10	1.56	.07	.123	[-.03,.24]	.15
	Immorality + Amusement	104	2.73	.81	2.76	1.09	.03	.48	.07	.630	[-.10,.17]	.04
Negative	No-Treatment Control	113	1.42	.53	1.44	.64	.02	.52	.03	.600	[-.05, .07]	.06
	**Immorality**	**115**	**1.56**	**.69**	**2.17**	**.81**	**.61**	**8.64**	**.07**	**< .001**	**[.47,.75]**	**.81**
	**Immorality + Kindness**	**115**	**1.57**	**.69**	**1.67**	**.67**	**.10**	**1.99**	**.05**	**.049**	**[.001,.20]**	**.18**
	Kindness	106	1.42	.55	1.47	.65	.04	.98	.04	.332	[-.05,.12]	.12
	**Amusement**	**112**	**1.53**	**.67**	**1.27**	**.51**	**-.26**	**-6.71**	**.04**	**< .001**	**[-.33,-.18]**	**.64**
	Immorality + Amusement	104	1.66	.70	1.56	.60	-.09	-1.58	.06	.117	[-.21,.02]	.17

*SD* = Standard Deviation. *SE* = Standard Error. CI = Confidence Intervals. Bold denotes a significant effect.

### Elevation, perceptions of humanity, and pro-sociality after reading the news stories

To examine the effect of condition on elevation, perceptions of humanity, and pro-sociality we conducted three ANOVAs, one per dependent variable. There was a significant effect of condition on elevation (*F*(5, 659) = 94.73, *p* < .001, η^2^ = .42) and perceptions of humanity (*F*(5, 659) = 5.73, *p* < .001, η^2^ = .04), but not pro-sociality (*F*(5, 659) = 1.30, *NS*, η^2^ = .01). As shown in Table 10, the highest levels of elevation and most positive perceptions of humanity were reported by participants in the Kindness condition.

**Table 10:** Study 2: Means and standard deviations for elevation, perceptions of humanity, and pro-sociality.

Condition	Elevation		Perceptions of Humanity		Pro-sociality	
	Mean	*SD*	Mean	*SD*	Mean	*SD*
No-Treatment Control	4.70	1.36	3.96	.72	£36.58	17.71
Immorality	2.81	1.08	3.56	.83	£33.45	19.58
Immorality+Kindness	6.24	1.53	3.93	.85	£39.23	20.09
Kindness	6.75	1.58	4.09	.86	£36.95	22.69
Amusement	4.50	1.84	4.01	.79	£36.65	19.28
Immorality+Amusement	4.43	1.73	3.86	.81	£39.22	19.68

SD = Standard Deviation

### Can kindness alleviate the aversive effects of reading about others’ immorality?

Planned contrasts showed that participants in the Immorality+Kindness condition fared significantly better than participants in the Immorality condition (see Table 11). Specifically, compared to participants in the Immorality only condition, they experienced significantly greater increases in positive affect, significantly smaller increase in negative affect (see Fig 3), and were significantly more likely to believe in the goodness of others and to display more pro-sociality.

**Table 11:** Study 2: Examining the effects of the immorality condition vs. The Immorality+Kindness condition on changes to affect, elevation, perceptions of humanity and pro-sociality.

Outcome Variable	Immorality + Kindness		Immorality		Planned Contrasts					
	Mean	*SD*	Mean	*SD*	Mean difference	*SE*	*t*	*p*	95% CI	*d*
Change in PA	**.53**	**.68**	**-.49**	**.64**	**1.02**	**.09**	**11.75**	**< .001**	**[.85, 1.19]**	**1.55**
Change in NA	**.10**	**.54**	**.61**	**.75**	**-.51**	**.09**	**-5.85**	**< .001**	**[-.68, -.34]**	**-.78**
Elevation	**6.24**	**1.53**	**2.81**	**1.08**	**3.43**	**.17**	**19.61**	**< .001**	**[3.08, 3.77]**	**2.59**
Humanity	**3.93**	**.85**	**3.56**	**.83**	**.37**	**.11**	**3.42**	**< .001**	**[.16, .58]**	**.43**
Pro-sociality	**£39.23**	**20.09**	**£33.45**	**19.58**	**5.78**	**2.62**	**2.21**	**.028**	**[.64, 10.93]**	**.39**

PA = Positive Affect. NA = Negative Affect. *SD* = Standard Deviation. *SE* = Standard Error. CI = Confidence Intervals. Bold denotes a significant effect.

### Are light-hearted news stories as effective as kindness news stories?

We next examined whether news stories featuring amusement would be as effective as kindness in alleviating the adverse effects of exposure to others’ immorality. We first confirmed that amusement varied between the conditions by conducting a one-way ANOVA which showed that condition had a significant effect on amusement, *F*(5, 659) = 119.20, *p* < .001, η^2^ = .48. Following up with planned contrasts we found that, as intended, the Amusement condition was rated as significantly more amusing than the other conditions (see Table 12).

**Table 12:** Study 2: Means and standard deviations for amusement per condition.

Condition	Mean	SD	Comparison to Amusement Condition
Amusement	6.29	1.86	-
No-Treatment Control	4.16	1.79	*t*(223) = 8.74, *p* < .001, CI95 = [1.64, 2.60]
Immorality	1.30	.76	*t*(225) = 26.33, *p* < .001, CI95 = [4.61, 5.36]
Immorality+Kindness	3.07	1.52	*t*(225) = 14.23, *p* < .001, CI95 = [2.77, 3.66]
Kindness	3.66	1.73	*t*(216) = 10.77, *p* < .001, CI95 = [2.14, 3.10]
Immorality+Amusement	5.19	2.13	*t*(214) = 3.99, *p* < .001, CI95 = [.55, 1.62]

*SD* = Standard Deviation. CI = Confidence Interval.

Relative to participants in the Immorality condition, participants in the Immorality+Amusement condition reported significantly higher levels of positive affect, elevation, and positive perceptions of humanity, and lower levels of negative affect (see Table 13 and Fig 3).

**Table 13:** Study 2: Relative effectiveness of Immorality+Kindness and Immorality+Amusement in their impacts on changes to affective well-being, elevation, perceptions of humanity, and pro-sociality.

	Planned Contrasts: Immorality vs. Immorality+Kindness						Planned Contrasts: Immorality vs. Immorality+Amusement					
	*Mean Difference*	*SE*	*t*	*p*	95% CI	*d*	*Mean Difference*	*SE*	*t*	*p*	95% CI	*d*
Change in PA	**1.02**	**.09**	**11.75**	**< .001**	**[.85, 1.19]**	**1.55**	**.52**	**.09**	**5.80**	**< .001**	**[.35, .70]**	**.79**
Change in NA	**-.51**	**.09**	**-5.85**	**< .001**	**[-.68, -.34]**	**-.78**	**-.70**	**.09**	**-7.64**	**< .001**	**[-.88, -.52]**	**-1.02**
Elevation	**3.43**	**.17**	**19.61**	**< .001**	**[3.08, 3.77]**	**2.59**	**1.62**	**.20**	**8.21**	**< .001**	**[1.24, 2.00]**	**1.14**
Humanity	**.37**	**.11**	**3.42**	**< .001**	**[.16, .58]**	**.43**	**.30**	**.11**	**2.69**	**.007**	**[.07, .52]**	**.36**
Pro-sociality	**5.78**	**2.62**	**2.21**	**.028**	**[.64, 10.93]**	**.39**	**5.77**	**2.69**	**2.15**	**.032**	**[.53, 11.00]**	**.29**

PA = Positive Affect. NA = Negative Affect. *SD* = Standard Deviation. *SE* = Standard Error. CI = Confidence Intervals. Bold denotes a significant effect.

Amusement was more effective than kindness at mitigating the negative effect of exposure to immorality for negative affect (respectively, *d* = -1.02 vs. *d* = -.78). Kindness was more effective than amusement at mitigating reductions in positive affect after exposure to others’ immorality (*d* = 1.55 vs. *d* = .79), inducing elevation (*d* = 2.59 vs. *d* = 1.14), promoting positive perceptions of humanity (*d* = .43 vs. *d* = .36), and promoting pro-sociality (*d* = .39 vs. *d* = .29).

## Discussion

Study 2 provided further evidence that news stories featuring others’ kindness can alleviate the aversive effects of news stories featuring others’ immorality as despite utilising more diverse stimuli, changing the ratio of bad:good from 1:5 to 1:3, and having participants read rather than watch news stories, we obtained the same results as in Studies 1a-d. Beyond this, we also found that participants in the Immorality+Kindness condition were more inclined to act pro-socially, allocating an average of £5.78 more to others, compared to participants in the Immorality condition.

As in Studies 1a-d, we once again found that the aversive effects of immorality were alleviated rather than eradicated. Furthermore, we replicated findings from Study 1d suggesting that news stories featuring kindness may still have benefits above and beyond news stories provoking amusement. Although in Studies 1a-d, we found that the kindness news stories increased both positive and negative affect, in Study 2, the kindness news stories only significantly increased positive affect. This suggests exposure to “catastrophe compassion” [10, 45] type news stories may generate more mixed affective responses than exposure to news-stories simply featuring others’ kindness.

## General discussion

### Seeing others’ kindness alleviates the aversive effects of seeing others’ evil

In the present research we set out to address an intriguing but hitherto unaddressed question–can the aversive effects of news stories featuring the worst of humanity be subsequently alleviated through exposure to news stories featuring the best of humanity? Across Studies 1 and 2, using 5 separate samples, we consistently found that exposure to others’ kindness helped alleviate the aversive emotional and cognitive consequences of exposure to others’ acts of immorality. Specifically, participants that were exposed to both others’ immorality and others’ kindness experienced less aversive changes to their mood, reported higher levels of elevation, and held more positive perceptions of humanity than participants exposed only to others’ immorality. Moreover, in general, kindness was more effective than amusement in alleviating the aversive effects of exposure to others’ immorality, suggesting that there is something especially powerful about it, beyond it simply provoking affectively pleasant feelings. Such findings contribute to the growing body of research evidencing the bad effects of bad news [5, 10, 16–18], while also demonstrating the restorative “feel good” properties of subsequently seeing others’ kindness, which may offer an emotional reset [29]. More broadly the results are consistent with the idea that positive emotions can undo the effects of negative emotions [68]. We did, however, find that not all positive emotional experiences were equally effective; we found that elevation was more effective at undoing than amusement.

Importantly, seeing others’ kindness allowed participants to hold on to beliefs about the goodness of others–something the immorality focused news stories threatened. This was evident not only in participants’ perceptions of humanity scores, but also in their open text responses which often described their faith in mankind being restored (e.g., “*I still feel that we’re fundamentally decent–well most of us*. *And that’s worth clinging to”; “It makes me happy to see that there are people in the world we can trust and rely on*”). Given the associations between well-being and believing in the goodness of humanity, it is evident that seeing others’ kindness highlighted in the media can be beneficial for individuals. It may also prove beneficial for society more broadly, as when people see someone model pro-social behaviour they tend to act more pro-socially themselves [65]. Indeed, many participants voiced inspiration or a desire to do something good (“*I’m going to try and do selfless good deeds*”; “*I felt inspired to do better as a person and human being*”). Moreover, participants exhibited greater pro-social tendencies in the Immorality+Kindness condition relative to the Immorality only condition.

### Alleviation not eradication: The limits of exposure to others’ kindness following others’ immorality

In both Studies 1 and 2 we found that media exposure to others’ kind acts alleviated, rather than eradicated the aversive effects of other’s immorality, as despite experiencing elevation and more positive perceptions of humanity, participants still reported significant increases in negative affect. Moreover, participants in the Immorality+Kindness condition often shared in their own words that they had experienced bittersweet reactions to the news stories. For example, feeling sad but amazed/proud, or disgusted but inspired. Such mixed affective reactions are not uncommon, and previous researchers have demonstrated that it is possible for oppositely valenced emotions to co-occur [69, 70]. For instance, in the case of nostalgia, good feelings are elicited through positive memories, but bad feelings because those times have been and gone [71, 72].

### Simply seeing others’ kindness can evoke both positive and negative reactions

Interestingly, even in the Kindness only conditions, participants also experienced mixed affective responses. This was particularly the case in Studies 1a-d, where the Kindness clips featured citizens helping out in the aftermath of terrorist attacks, but was also apparent in Study 2, in participants’ open text responses. One prevalent source of negativity was sadness that help was needed in the first place (“*Felt good that there are people out there that help others selflessly*, *but also sad mixed with a bit of despair knowing it was necessary in the first place*”; “*They were lovely stories which made me feel happy that people were going out their way to help others but sad that problems exist”*). In some cases this presented itself as frustration at various institutions for not taking ownership of societal challenges (e.g., “*Whilst it is nice to see what these people are doing I feel that they are ultimately performing roles which the state should be to some extent*. *So although it is nice on the one hand it is also sad that the services aren’t available to help*”). A considerable number of participants also reported feelings of relative prosocial inadequacy (e.g., “*I feel I could be doing more as a human being for others”*; “*It made me feel sad I don’t*, *or can’t*, *do more myself*”). Such responses appear aligned with one aspect of elevation–“the desire to be a better person” [30] which indirectly alludes to the idea of feeling like a worse person. Feelings of being shamed by others’ kindness may also speak to the desire not only to see others being helped [49, 57], but to be the one doing the helping. This may be motivated by a desire to act in a way that is highly valued personally and globally [31, 32], and/or simply because helping boosts happiness [41, 55, 56].

Overall, while unanticipated, these complex and diverse reactions to seeing others’ kindness contribute to the literature, which has predominantly perceived viewing others’ acts of kindness and the resulting feelings of elevation as unambiguously positive [28, 29, 59, 60]. Clearly further research is needed to better understand this potentially darker side to seeing others’ kindness.

### Real world implications

In light of the considerable evidence documenting the negative impacts of negatively framed stories on mental health and intentions to undertake positive societal actions, our findings indicate that there is merit in adopting a balanced journalistic perspective that does not solely highlight the worst of humanity in a bid to captivate readers [5, 18, 45]. It seems there is something especially powerful about showing others’ kindness in the aftermath of others’ immorality and we encourage news outlets to adopt this format where possible.

More generally, we follow other researchers in calling for the media to include a more balanced coverage incorporating some positively valenced content [18]. This is not to say that news-stories should be substanceless fluff, or that media coverage should use positive stories to distort realities or gloss over institutional failings. Rather, we seek to encourage further consideration of alternative ways to cover serious topics without inciting aversive reactions through intentionally provocative and emotive sensationalism. For instance researchers have begun to explore the pros and cons of constructive journalism where news stories use solution-oriented framing [5]. Such an approach involves journalists rigorously reporting social issues but also how people and society are responding to them [24]. This allows important issues to be covered without inciting apathy and hopelessness or even compassion collapse, an effect whereby people become numb to others’ suffering [19].

Another approach that warrants further empirically-based investigation is the use of satirical news (i.e., comedic commentary on real news stories such as “Have I Got News for You or The Colbert Show). Indeed, our own results provide some preliminary indication that amusement, though not as effective as kindness, may also prove helpful in dispelling some of the emotional and cognitive disturbances caused by exposure to negatively valenced news. Moreover, preliminary research suggests satirical news may be effective in capturing the attention of people who would otherwise avoid engaging with political news [73].

## Conclusion

Our empirical findings support the notion of presenting the best of humanity alongside the worst of humanity in media coverage as people are less likely to suffer from emotional disturbances and an increased sense that the world they live in is a dark and dangerous place [6, 38]. After all, perceiving humanity as good reinforces notions that just as we value benevolence, so too do others in the world [31, 32]. Such beliefs appear important for our emotional well-being [33], and our willingness to participate in and contribute to society, including the extent to which we are willing to engage in philanthropy or even perform eco-oriented actions [5]. Given this, we urge future research to further explore the conditions under which seeing others’ kindness may benefit individuals and society, and to further establish the case for journalists to shine a spotlight on others’ kindness.
